# Is Ferroptosis the Mechanistic Bridge Connecting Iron Dysregulation to Muscle Wasting and Functional Decline in Aging?

**DOI:** 10.1111/acel.70367

**Published:** 2026-01-14

**Authors:** Rola S. Zeidan, Simon Reinhard, Anna Picca, Emanuele Marzetti, Christiaan Leeuwenburgh, James F. Collins, Stephen D. Anton

**Affiliations:** ^1^ Department of Physiology and Aging, College of Medicine University of Florida Gainesville Florida USA; ^2^ Department of Health Outcomes and Biomedical Informatics College of Medicine, University of Florida Gainesville Florida USA; ^3^ Department of Medicine and Surgery LUM University Casamassima Italy; ^4^ Fondazione Policlinico Universitario “Agostino Gemelli” IRCCS Rome Italy; ^5^ Department of Geriatrics, Orthopedics and Rheumatology Università Cattolica del Sacro Cuore Rome Italy; ^6^ Food Science and Human Nutrition Department Institute of Food and Agricultural Sciences, University of Florida Gainesville Florida USA

**Keywords:** Ferroptosis, iron, older adults, physical function, skeletal muscle

## Abstract

Age‐related decline in physical function is a hallmark of aging and a major driver of morbidity, disability, and loss of independence in older adults, yet the molecular processes linking muscle aging to functional deterioration remain incompletely defined. Emerging evidence implicates ferroptosis, defined as iron‐dependent, lipid peroxidation‐driven cell death, as a compelling but underexplored contributor to age‐related muscle wasting and weakness. Although ferroptosis signatures appear in aged muscle across cellular, animal, and human studies, their causal role in functional decline has not been clearly established. Here, we synthesize current evidence to propose a framework in which iron dyshomeostasis, impaired antioxidant defenses, and dysregulated ferritinophagy converge to create a pro‐ferroptotic milieu that compromises muscle energetics, structural integrity, and regenerative capacity. We delineate key knowledge gaps, including the absence of ferroptosis‐specific biomarkers in human muscle and limited longitudinal data linking ferroptotic stress to mobility outcomes. Finally, we highlight potential therapeutic opportunities targeting iron handling and lipid peroxidation pathways. A better understanding of the contribution of ferroptosis to muscle aging may enable development of mechanistically informed biomarkers and interventions to preserve strength and mobility in older adults.

## Graphical abstract

This work proposes ferroptosis as a mechanistic driver of age‐related muscle dysfunction, whereby iron dyshomeostasis and impaired antioxidant capacity promote lipid peroxidation, and eventually trigger ferroptosis, leading to muscle wasting, and ultimately contributing to weakness and functional decline with aging.

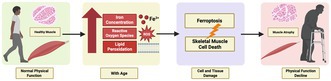

## 1 Introduction

Decline in physical function is a key health concern for older adults, since it can lead to loss of independence, increased morbidity, and reduced quality of life (Aubert et al. 2022). Extensive evidence demonstrates that muscle wasting in older adults is strongly associated with declines in strength, mobility, and overall physical function, contributing to frailty and loss of independence (Cruz‐Jentoft et al. 2019). Therefore, elucidating the molecular mechanisms underlying muscle loss may provide critical insights into the biological pathways driving physical function decline in older adults.

Skeletal muscle, which accounts for approximately 40% of total body weight (Janssen et al. 2000), plays a central role in mobility, metabolic regulation, and overall health, and is therefore particularly vulnerable to age‐related dysfunction (Aubert et al. 2022). Muscle wasting in aging is not only a direct driver of frailty and disability but is also linked to chronic conditions whose risk increases with age, such as type 2 diabetes mellitus, immune dysfunction, and chronic kidney disease (Cheng et al. 2022; Janssen et al. 2000; Tsekoura et al. 2017). Advancing our understanding of the pathogenesis of age‐associated muscle wasting is a pressing medical concern since this knowledge can facilitate the development of therapeutic strategies to treat or prevent these conditions.

Emerging evidence indicates a role for ferroptosis, a distinct form of regulated cell death driven by iron‐dependent lipid peroxidation, as a potential contributor to age‐related muscle atrophy and consequent physical function decline (Wang, Zhang, et al. 2022). Unlike general oxidative stress, ferroptosis can be specifically identified by its requirement for iron, sensitivity to glutathione peroxidase 4 (GPX4) inactivation or glutathione (GSH) depletion, rescue by ferroptosis inhibitors such as ferrostatin‐1 or liproxstatin‐1, and morphological features including condensed mitochondria with reduced cristae, while remaining independent of apoptosis or necroptosis pathways (Yan, Zou, et al. 2021; Yang and Stockwell 2016). Noteworthy, GSH and GPX4 form a critical defense against ferroptosis, with GPX4 using GSH as a cofactor to reduce lipid hydroperoxides and prevent iron‐dependent membrane damage. Accordingly, depletion of GSH or inhibition of GPX4 can trigger ferroptotic cell death (Ursini and Maiorino 2020).

Consistent with this framework, multiple ferroptosis‐related molecular pathways intersect with pathological processes implicated in skeletal muscle atrophy and motor neuron loss (Cook and Yu 1998; Wang, Zhang, et al. 2022), both of which contribute to physical function decline. This convergence suggests a mechanistic link between iron dysregulation and impaired physical function, particularly in older adults, where disrupted homeostatic responses are prominent and can amplify vulnerability to tissue damage (Alves et al. 2023). Indeed, with advancing age, skeletal muscle exhibits progressive cellular iron accumulation, which promotes oxidative stress, mitochondrial dysfunction, and lipid peroxidation (DeRuisseau et al. 2013; Tounaoua et al. 2025), all hallmark features of ferroptotic damage. These cellular events contribute to impaired protein turnover, leading to reduced protein quality control and myofiber integrity (Halon‐Golabek et al. 2019). This eventually leads to loss of muscle fibers and reduced contractile function, providing a plausible biological basis for the observed association between elevated iron stores and age‐related physical function decline driven by muscle mass loss (Sun et al. 2024).

**FIGURE 1:**
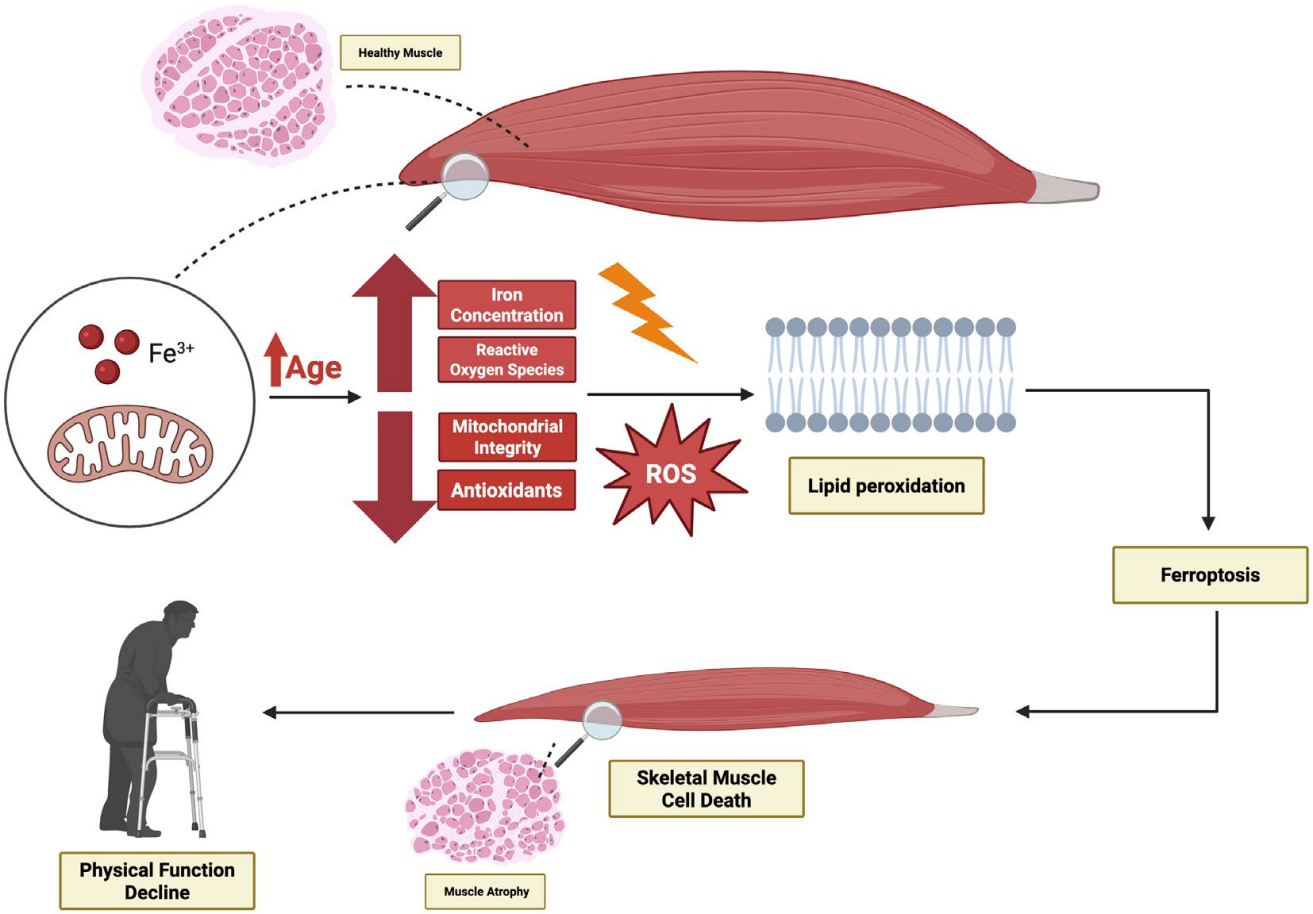
Proposed mechanism linking aging to skeletal muscle degeneration via ferroptosis. This schematic illustrates a potential mechanism by which aging may contribute to skeletal muscle atrophy and functional decline through ferroptosis. Aging is associated with increased iron accumulation, elevated reactive oxygen species (ROS), and reduced mitochondrial integrity and antioxidant defenses. These changes may promote excessive ROS production, leading to lipid peroxidation of cellular membranes. The resulting oxidative damage may trigger ferroptosis, an iron‐dependent form of regulated cell death. This process could contribute to skeletal muscle cell death, muscle atrophy, and the progressive decline in physical function observed with aging.

Notably, approximately 10%–15% of total body iron is found in skeletal muscle, primarily bound in myoglobin and mitochondrial enzymes that sustain energetic demands (Ordway and Garry 2004). Although ferritin serves as the universal iron storage protein, the enrichment of iron‐containing proteins in muscle underscores its dependence on iron for normal function (Beard 2001). This substantial iron content, combined with abundant mitochondria, renders skeletal muscle particularly vulnerable to iron‐mediated oxidative damage and ferroptosis (Figure 1). Although the study of ferroptosis is still in an emerging stage, understanding its potential role in muscle wasting and age‐associated conditions that can impair physical function (such as osteoarthritis) is crucial for developing targeted interventions to mitigate decline in older adults. In this review, we synthesize current evidence connecting iron dysregulation, oxidative stress, and regulated cell death pathways in aging skeletal muscle, with a particular focus on ferroptosis. The novelty of this work lies in linking ferroptotic mechanisms to age‐associated muscle mass and functional decline. It is important to note, however, that most mechanistic insights into ferroptosis in muscle are derived from animal or cell‐based studies. Direct evidence demonstrating bona fide ferroptotic cell death in human skeletal muscle remains limited, largely because current biomarkers lack specificity for ferroptosis relative to general oxidative injury. Thus, while ferroptosis represents a biologically plausible model, its causal contribution to human muscle aging remains an active area of investigation.

## 2 Mechanistic Drivers of Ferroptosis in Muscle Wasting

The mechanistic events described below reflect experimentally supported ferroptosis pathways characterized primarily in preclinical systems. While these pathways provide a strong conceptual framework for understanding how iron‐driven lipid peroxidation may contribute to muscle atrophy, definitive demonstration of these mechanisms in human muscle tissue remains challenging due to the absence of ferroptosis‐specific readouts.

### 2.1 Iron Dysregulation and Lipid Peroxidation

Age‐associated iron dysregulation is a main driver of ferroptosis, with cellular iron overload being its defining feature (Zeidan et al. 2021). As such, when intracellular ferrous iron (Fe^2+^) is elevated, iron is more likely to react with hydrogen peroxide in the so‐called Fenton reaction, leading to the generation of hydroxyl radicals that trigger peroxidation of cell membrane lipids (Jingsong et al. 2024). The accumulation of 4‐hydroxy‐2‐nonenal (4‐HNE), a toxic byproduct of lipid peroxidation, exacerbates cellular damage and promotes ferroptosis (Sebastian et al. 2016). Under physiological conditions, antioxidant systems such as GSH, GPX4, superoxide dismutase (SOD), and catalase reduce lipid peroxidation and prevent oxidative stress (Conrad et al. 2018; Tang et al. 2021). Early preclinical evidence in models of aging and muscle disuse indicates that reduced expression of ferroportin (the sole cellular iron exporter) leads to increased iron sequestration, elevated oxidative stress, and thereby exacerbated muscle damage (Hofer et al. 2008; Xu et al. 2008, 2012). With aging, increased susceptibility to oxidative stress and weakened antioxidant defenses promote ferroptosis, resulting in loss of myofibers and impaired mitochondrial bioenergetics, ultimately leading to skeletal muscle weakness and reduced physical function (Sun et al. 2024). However, these findings have not yet been examined in the context of ferroptosis, leaving the mechanistic links between iron retention and regulated cell death unresolved.

A study by Eshima et al. (2023) in human and murine skeletal muscle samples revealed that age‐associated accumulation of reactive oxygen species (ROS) results in elevation of lipid hydroperoxides, which are further exacerbated by advanced age and reduced physical function‐associated GPX4 reduction, leading to muscle atrophy. Notably, lipid hydroperoxides generate secondary reactive lipid aldehydes, such as 4‐HNE and malondialdehyde (MDA), that cause carbonyl stress and readily react with cellular biomolecules, leading to cellular toxicity and damage (Ayala et al. 2014; Eshima et al. 2023). Collectively, these findings indicate that the buildup of lipid hydroperoxides and their reactive aldehyde products, both of which are well‐established executors of ferroptotic death, likely contribute to pro‐ferroptotic conditions in aging muscle. Therefore, they may serve as both molecular markers and drivers of ferroptosis, contributing to the progressive age‐related loss of muscle mass and function (Dixon et al. 2012; Kagan et al. 2016). Together, these processes create a self‐perpetuating cycle of oxidative stress that drives organelle dysfunction and progressively undermines muscle resilience with age.

### 2.2 Mitochondrial Dysfunction and Impaired Antioxidant Defense

Mitochondria contain a substantial proportion of intracellular iron (up to 20%–50%), reflecting their high demand for iron‐containing proteins involved in energy production, heme synthesis, and iron–sulfur cluster assembly (Jhurry et al. 2012; Paul et al. 2016). Mitochondrial iron mainly participates in iron–sulfur (Fe–S) cluster biogenesis, heme synthesis, and energy production via the electron transport chain, with the majority of mitochondrial iron metabolism taking place within the mitochondrial matrix (Ben Zichri‐David et al. 2025). Perhaps also due to containing substantial amounts of intracellular iron, mitochondria serve as the main intracellular producers of ROS (Dan Dunn et al. 2015). Recent studies indicate that mitochondrial dynamics, including fission and fusion, and mitophagy, influence iron distribution and ROS production, creating a bidirectional relationship that affects susceptibility to ferroptosis (Li et al. 2023; Pedrera et al. 2025; Zhou, Ren, et al. 2024).

During ferroptosis, mitochondrial ROS, in the presence of redox‐active iron, catalyze the peroxidation of polyunsaturated fatty acids (PUFAs) in cell membrane phospholipids (Barrera et al. 2016). Free PUFAs are substrates for the synthesis of lipid‐signaling mediators; however, to act as signals for ferroptosis, PUFAs must first be incorporated into membrane phospholipids (Kagan et al. 2016). Specific lipoxygenases (LOXs), which are nonheme iron‐dependent dioxygenases, can directly oxygenate PUFAs within biological membranes and thus be potential mediators of ferroptosis (Xuejun et al. 2021). Additionally, in aging muscle cells, mitochondrial dysfunction, specifically impaired electron transport and reduced capacity for iron–sulfur cluster biogenesis, further disrupts the antioxidant defenses, including the cystine/glutamate antiporter (System Xc−), GSH, and GPX4, thereby promoting ferroptotic cell death (Lei et al. 2022).

### 2.3 Inflammation and Cellular Senescence

**FIGURE 2:**
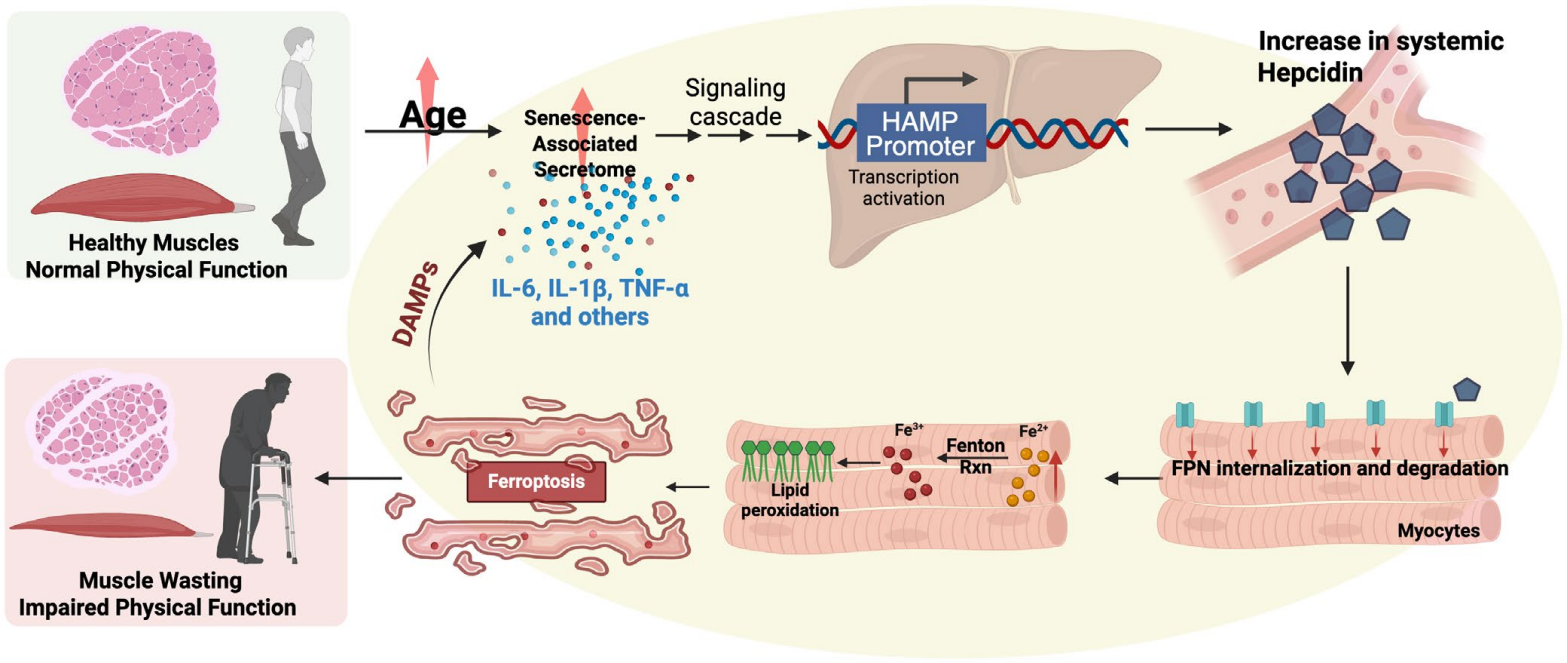
Inflammatory induction of hepcidin links senescence to ferroptosis in skeletal muscle. Senescent macrophages and other SASP‐secreting cells release proinflammatory cytokines (IL‐6, IL‐1β, TNF‐α) that act on the liver and local stromal cells to activate the HAMP promoter, inducing hepcidin synthesis. Hepcidin binds to ferroportin (FPN1) on myocytes and other cells (such as macrophages and enterocytes), triggering its internalization and lysosomal degradation, thereby blocking iron export. The resulting accumulation of redox‐active ferrous iron (Fe^2+^) promotes Fenton chemistry, generating reactive oxygen species (ROS) that drive lipid peroxidation and ferroptotic cell death. Ferroptotic muscle fibers release damage‐associated molecular patterns (DAMPs) that further amplify systemic inflammation and the SASP, creating a feed‐forward loop that sustains hepcidin elevation and tissue iron retention.

In addition to age‐associated cellular iron accumulation and mitochondrial dysfunction, several systemic processes converge to exacerbate redox imbalance in aging muscle. Among these, chronic low‐grade inflammation (or inflammaging) emerges as a strong risk factor for many age‐related diseases, including sarcopenia and neurodegeneration (Antuña et al. 2022; Arsun et al. 2017). While inflammation is essential for the maintenance and repair of organs, persistent activation of inflammation, however, drives muscle wasting through sustained cytokine release and cellular senescence (Pawelec et al. 2014; Wang et al. 2017). Growing evidence shows that dysregulated inflammatory signaling can enhance iron imbalance and lipid peroxidation, thereby promoting ferroptosis and contributing to tissue dysfunction (Chen, Fang, et al. 2023). Proinflammatory cytokines, including TNF‐α and IL‐6, downregulate GPX4, a key ferroptosis regulator, promoting iron‐dependent lipid peroxidation and myofiber atrophy (Yitian et al. 2020). In parallel, inflammation induces the hepatic hormone and systemic iron regulator, hepcidin, which downregulates ferroportin, promoting iron accumulation within cells and thus generating a pro‐oxidative milieu that further enhances ferroptosis (Qin et al. 2023). Ferroptosis, in turn, amplifies inflammation by inducing the release of cytokines and damage‐associated molecular patterns (DAMPs) from dying cells, creating a feed‐forward loop of injury (Kim et al. 2018) (Figure 2). Senescent stromal cells further exacerbate this process by secreting senescence‐associated secretory phenotype (SASP) factors, which maintain a chronic inflammatory state (Campisi et al. 2007). In parallel, senescent macrophages can infiltrate skeletal muscle and promote ferroptosis, thereby exacerbating muscle atrophy (Xiang et al. 2025). Consistent with these mechanisms, elevated circulating pro‐inflammatory cytokines and C‐reactive protein are strongly linked to reduced muscle strength and mass, and a higher risk of sarcopenia (Schaap et al. 2006; Tuttle et al. 2020). Taken together, inflammaging, cellular senescence, and ferroptosis could lead to a vicious circle that accelerates skeletal muscle loss and physical function decline in older adults (Figure 2).

### 2.4 Endoplasmic Reticulum Stress, Proteostasis Collapse, and Ferritinophagy Activation

Aging muscle is characterized by a gradual decrease in proteostasis, which contributes to muscle loss (Shukla and Narayan 2025). This loss of proteostatic control is increasingly linked to endoplasmic reticulum (ER) stress and impaired autophagic (cytoprotective recycling pathway) flux, two interconnected processes that amplify ferroptotic vulnerability in muscle (Guo et al. 2024; Han et al. 2025). Chronic ER stress activates the unfolded protein response (UPR), increasing oxidative load and lipid peroxidation while depleting glutathione and suppressing GPX4 activity (Guo et al. 2024; Zhou, Wei, et al. 2024). In parallel, age‐related activation of ferritinophagy (a selective autophagic Nuclear Receptor Coactivator 4 (NCOA4)‐mediated process that targets ferritin for lysosomal degradation) leads to uncontrolled iron release from ferritin into the labile iron pool (Di Lorenzo et al. 2025; Liu et al. 2022). This excess iron accelerates Fenton chemistry and lipid radical propagation at mitochondrial and ER membranes, disrupting proteostasis and mitochondrial dynamics, thus compromising cellular bioenergetics and structural integrity (Dixon et al. 2012; Wang, Zhang, et al. 2022). The resulting mitochondrial dysfunction leads to loss of bioenergetic reserve, which directly impairs muscle contractility and fatigue resistance, thereby linking cellular events to measurable physical decline (Chen, Ji, et al. 2023; Kubat et al. 2023; Romanello and Sandri 2015). Notably, as lysosomal clearance efficiency declines with age, partially degraded ferritin and oxidized lipids accumulate, sustaining lipid peroxidation‐driven organelle stress and ferroptotic cell death (Chen, Kung, and Gnana‐Prakasam 2022; Guerrero‐Navarro et al. 2022). Further, these processes lead to amplified pro‐senescent signaling through the SASP (Alves et al. 2023; Di Lorenzo et al. 2025). Collectively, these events propagate oxidative and inflammatory stress to neighboring fibers and macrophages, thereby altering muscle‐immune crosstalk and accelerating sarcopenic remodeling (Guo et al. 2022).

**FIGURE 3:**
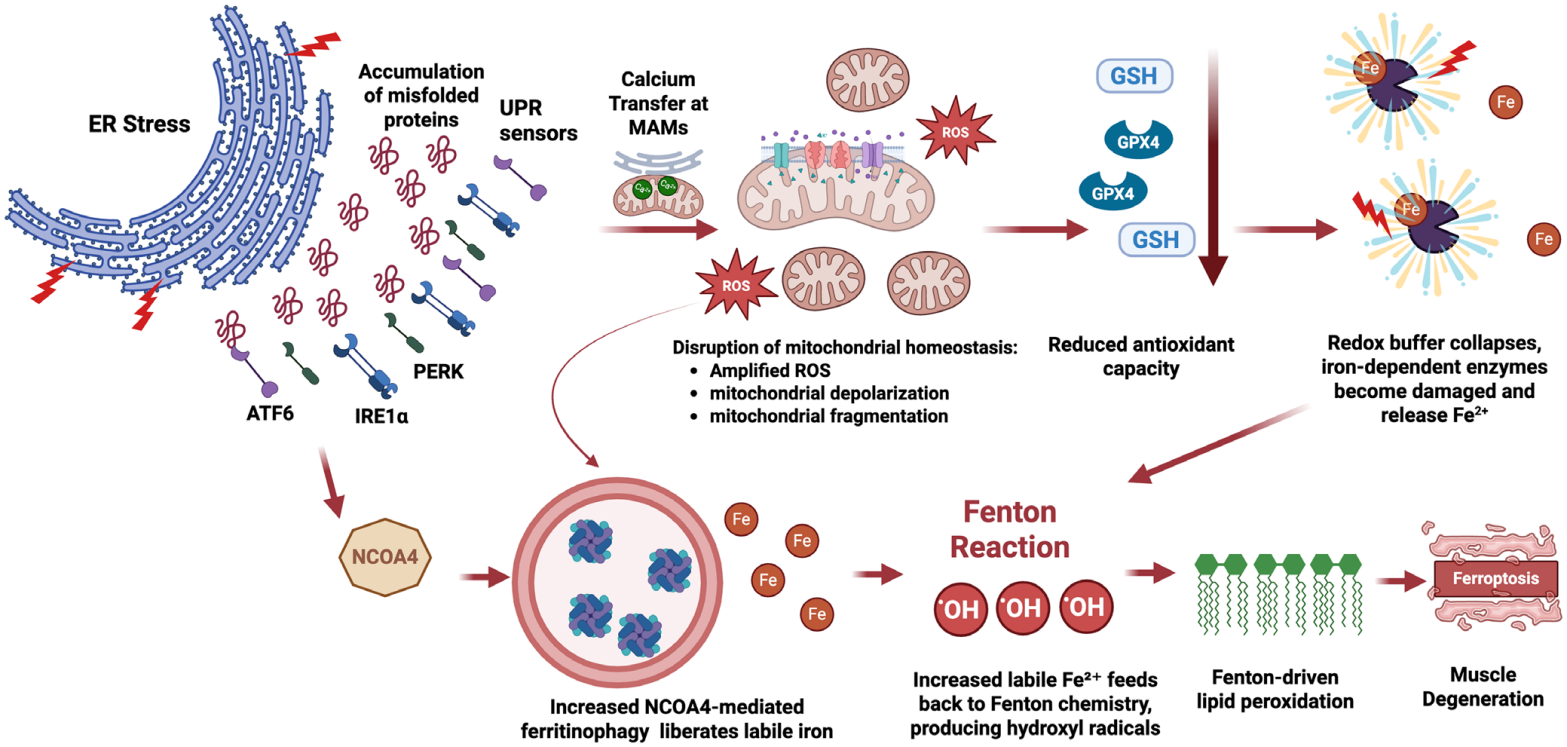
Convergence of endoplasmic reticulum stress, proteostatic collapse, and iron‐driven lipid peroxidation promotes ferroptotic muscle degeneration in aging. Persistent endoplasmic reticulum (ER) stress activates the unfolded protein response (UPR) sensors PERK, IRE1α, and ATF6, leading to the accumulation of misfolded proteins and sustained oxidative stress. UPR signaling at ER–mitochondria contact sites (mitochondria‐associated membranes‐MAMs) drives Ca^2+^ transfer, mitochondrial depolarization, and fragmentation, resulting in excessive mitochondrial reactive oxygen species (ROS) production and bioenergetic failure. The elevated ROS burden depletes the glutathione (GSH)–glutathione peroxidase 4 (GPX4) defense system, lowering antioxidant capacity and sensitizing membranes to lipid peroxidation. Concurrently, UPR‐induced activation of ATF4 and TFEB (not shown) upregulates NCOA4‐mediated ferritinophagy, releasing labile iron (Fe^2+^) from ferritin stores. The liberated iron catalyzes Fenton reactions, generating hydroxyl radicals (•OH) that exacerbate lipid peroxidation at ER and mitochondrial membranes. The convergence of UPR‐driven mitochondrial dysfunction, iron mobilization, and oxidative membrane injury culminates in ferroptotic cell death, leading to myofibrillar loss, impaired contractility, and progressive decline in muscle quality and physical function with aging.

Moreover, crosstalk between ER stress sensors (major signaling branches of UPR, including Protein Kinase RNA‐like Endoplasmic Reticulum Kinase‐PERK, Inositol‐Requiring Enzyme 1 alpha‐IRE1α, and Activating Transcription Factor 6‐ATF6) and mitochondrial ROS signaling exacerbates redox imbalance and suppresses adaptive mitophagy (Kumar and Maity 2021; Senft and Ronai 2015). The resulting convergence of proteotoxic and lipid‐peroxidative stress forms a “double‐hit” mechanism driving muscle fiber vulnerability (Paez et al. 2023) (Figure 3). The downstream collapse of proteostasis not only triggers ferroptotic death but also disrupts myofibrillar architecture, impairing muscle contraction and regeneration due to an imbalance between anabolic and catabolic processes (Fuqua et al. 2023). Collectively, ferroptosis‐associated disruption of ER‐mitochondria quality control creates a mechanistic bridge linking cellular iron accumulation to loss of muscle integrity and physical function with age. As proteostasis and ferritinophagy progressively fail, iron and oxidative damage accumulate, accelerating myofiber atrophy and mobility decline in older adults.

## 3 Regenerative Failure in Aging Muscle: Satellite Cells, Iron, and Ferroptosis

Satellite cells, which are the adult muscle stem cells essential for postnatal growth and regeneration, exhibit a distinct sensitivity to disruptions in iron metabolism (Fujimaki et al. 2016; Ru et al. 2025). As in most other cells, iron is required for key satellite cell functions, including mitochondrial respiration, DNA synthesis, and cell‐cycle progression (Halon‐Golabek et al. 2019; Zeidan et al. 2021). Iron balance in skeletal muscle is controlled primarily by the iron‐regulatory protein/iron‐responsive element (IRP/IRE) system, which adjusts iron uptake and storage according to intracellular needs (Zeidan et al. 2021). During iron deficiency, IRP1/2 increase the stability and translation of iron‐import genes such as Tfr1 while suppressing ferritin synthesis, thereby enhancing iron availability for mitochondrial metabolism and cell‐cycle progression (Hentze et al. 2010; Rouault 2006). Iron homeostasis in satellite cells is essential for their function, and although direct mechanistic studies are still limited, current evidence suggests that precise iron regulation becomes particularly important during satellite‐cell activation, when metabolic iron requirements rise. Studies have shown that iron deficiency impairs satellite cell proliferation and myogenic differentiation through multiple mechanisms, including reduced mitochondrial ATP production, induced ferritinophagy, and epigenetic regulation and HIF‐2α stabilization, which activates a cell‐cycle brake (via increased Rb1 and reduced E2F activity) that blocks satellite cell proliferation and impairs muscle regeneration (Che et al. 2023; Fu et al. 2025; Ru et al. 2025). Similarly, deletion of ZIP13, a metal‐handling transporter involved in skeletal muscle homeostasis, resulted in reduced satellite cell abundance and impaired regulation of their activation state, highlighting its importance in maintaining the regenerative stem cell pool (Yoshigai et al. 2025). Conversely, evidence from preclinical models indicates that iron overload in satellite cells disrupts cellular iron and lipid metabolism, increases oxidative stress and ferroptosis, and impairs satellite cell proliferation and regenerative capacity (Ding et al. 2021). This indicates that iron dysregulation may hinder the normal quiescence‐to‐activation transition required for effective regenerative capacity. Potentially, because quiescent satellite cells naturally maintain low ROS levels and rely on strong antioxidant defenses, they are particularly sensitive to disruptions in iron homeostasis that elevate oxidative stress and lipid peroxidation, making them vulnerable to iron‐driven injury when redox balance is perturbed (Lian et al. 2022). Congruently, a recent study demonstrated that chronic, age‐associated inflammation disrupts the epigenetic maintenance of muscle stem cell quiescence, via repression of anti‐ferroptosis genes, thereby inducing ferroptosis in muscle stem cells and contributing to their functional decline (Blanc et al. 2025).

Although ferroptosis has not been directly demonstrated in satellite cells, studies show that aging and iron dysregulation promote lipid peroxidation, reduce antioxidant defenses such as GPX4 and NRF2, and impair satellite cell proliferation and regenerative capacity, suggesting that satellite cells may be vulnerable to ferroptotic stress when iron homeostasis is disrupted (Le Moal et al. 2017; Schmidlin et al. 2019). Moreover, systemic inflammatory states such as aging, cachexia, and metabolic disease, which increase IL‐6 and consequently hepcidin, can alter local iron availability within the skeletal muscle tissue and thus the satellite cell niche, further compromising the regenerative potential (Hofer et al. 2008; Picca et al. 2019; Wrighting and Andrews 2006). Collectively, these findings indicate that iron dyshomeostasis may impair muscle regeneration and maintenance not only by damaging mature myofibers but also by disrupting satellite cell function, potentially leading to muscle wasting. Together, this highlights an underexplored interface between ferroptosis biology and stem cell‐mediated muscle repair during aging.

## 4 Distinguishing Ferroptosis from Other Regulated Cell Death Pathways

Although regulated cell death pathways have been implicated in muscle aging, ferroptosis has emerged as a particularly compelling and mechanistically plausible contributor under conditions of iron dysregulation and oxidative stress, both of which are prominent in aging. It specifically represents a biochemically distinct, iron‐dependent form of non‐apoptotic death driven by uncontrolled polyunsaturated lipid peroxidation and failure of the GPX4‐GSH‐dependent antioxidant systems (Li, Cao, et al. 2020). Here we note that these features are unique to ferroptosis and are not shared by any other form of regulated cell death discussed below. Moreover, preclinical evidence indicates that ferroptosis inhibitors can rescue myocyte death in certain conditions, such as mechanical trauma, providing strong evidence for our proposed cell‐death mechanism (He et al. 2023).

### 4.1 Apoptosis

In contrast, evidence supporting the involvement of other regulated cell death pathways in aging myocytes remains limited. To contextualize their distinct roles, it is important to briefly outline the canonical mechanisms of apoptosis, necroptosis, and pyroptosis. Apoptosis is an energy‐dependent process driven by caspase activation, mitochondrial cytochrome c release, and nuclear fragmentation (Elmore 2007). Earlier studies (Dirks and Leeuwenburgh 2002; Wohlgemuth et al. 2010) linked aging‐related muscle atrophy to increased apoptotic signaling, including elevated caspase activity and cytochrome c release. However, these changes were modest and inconsistent across muscle types, suggesting apoptosis may contribute to selective myonuclear turnover rather than global myofiber loss. More recent evidence implicates iron accumulation, redox imbalance, lipid peroxidation, mitochondrial dysfunction and morphological changes, which are hallmarks not explained by apoptosis, as central drivers of sarcopenic degeneration, positioning ferroptosis as a more comprehensive model of regulated cell death in aging muscle.

### 4.2 Necroptosis

Necroptosis, on the other hand, represents a receptor‐mediated lytic death initiated by death receptor signaling and executed through Receptor‐Interacting Serine/Threonine Kinase 1 (RIPK1), RIPK3, and Mixed‐Lineage Kinase Domain‐like Protein (MLKL), which form a necrosome complex that permeabilizes the plasma membrane and induces cellular swelling (Galluzzi et al. 2017). Recent reviews proposed that necroptosis signaling, mediated by RIPK1/RIPK3 and MLKL, increases with age and may contribute to tissue degeneration (Royce et al. 2019; Yang et al. 2022). However, direct evidence in skeletal muscle remains sparse, as most data derive from non‐muscle tissues or systemic aging models, and no studies have yet demonstrated bona fide necroptotic execution (for instance, MLKL phosphorylation or membrane rupture) in naturally aged myofibers.

### 4.3 Pyroptosis

Pyroptosis, an inflammasome‐driven process involving gasdermin D‐mediated pore formation leading to cell swelling, plasma membrane rupture, and release of pro‐inflammatory cytokines, primarily contributes to inflammatory myopathies and immune activation, and less to non‐inflammatory sarcopenic degeneration (Javadov 2022; Shi et al. 2015). Recent studies have proposed a role for pyroptosis in mouse muscle aging, including evidence of NLRP3 inflammasome activation and inflammatory caspase signaling in aged muscle tissue (Jin et al. 2022; McBride et al. 2017), as well as identification of a TNF‐α‐caspase‐8‐caspase‐3‐GSDME axis contributing to age‐related muscle loss in animal models, which might also indicate crosstalk between apoptosis and pyroptosis (Wu et al. 2023). However, in the latter study, inhibiting pyroptosis only partially improved sarcopenia. Additionally, these findings remain largely correlative, with limited direct demonstration of gasdermin‐mediated pore formation or myofiber lysis in naturally aged muscle, underscoring the need for mechanistic validation and cell‐type‐specific studies to establish pyroptosis as a definitive driver of sarcopenia.

### 4.4 Synopsis

Although multiple regulated cell death pathways may participate in muscle aging, accumulated evidence indicates that ferroptosis is likely a major contributor driving age‐associated skeletal muscle atrophy. Its molecular features closely mirror the biochemical state of aged myofibers (Liu et al. 2020). Supporting studies in human and animal muscle demonstrate GPX4 downregulation, oxidized phospholipid buildup, and rescue by iron chelators (deferoxamine) or lipophilic antioxidants (ferrostatin‐1, liproxstatin‐1) (Eshima et al. 2023; Yan, Beiling, et al. 2021; Ding et al. 2021; Czyżowska et al. 2023). In contrast, apoptosis, necroptosis, and pyroptosis appear less consistently activated and are mainly implicated in acute or inflammatory injury rather than chronic age‐related atrophy. Nonetheless, the potential coexistence or interaction of these pathways warrants further investigation.

## 5 Defining Ferroptosis Beyond Oxidative Stress

Given the growing mechanistic evidence that ferroptosis underlies structural and functional decline in aging muscle, the next challenge is to translate these molecular signatures into measurable biomarkers that can distinguish ferroptosis from generalized oxidative stress and other forms of cellular injury. Directly testing ferroptosis inhibitors in human muscle remains technically and ethically challenging, making biomarker discovery essential for inferring ferroptotic activity in vivo. From a translational standpoint, ferroptosis can be analytically distinguished from general oxidative stress through its biochemical, morphological, and pharmacological signatures. Candidate biomarkers include circulating ferritin, 4‐HNE, MDA, and muscle‐specific transcripts such as decreased GPX4 or SLC7A11 and elevated Acyl‐CoA Synthetase Long‐Chain Family Member 4 (ACSL4‐which enriches cellular membranes with polyunsaturated phospholipids that serve as substrates for iron‐dependent lipid peroxidation), or NCOA4 (Chen et al. 2021). Integrating these markers with functional assays of mitochondrial function, morphology, and redox homeostasis may enable the in vivo identification of ferroptotic activity in aging muscle. Emerging human data (Table 1) demonstrate that ferroptosis‐related biomarkers correlate with mobility and strength measures, strengthening the biological plausibility that ferroptosis contributes directly to physical function decline. Establishing and validating such biomarkers will be pivotal for distinguishing ferroptosis from nonspecific oxidative injury. This will also be important for developing targeted iron‐modulating, Nuclear Factor Erythroid 2–Related Factor 2 (NRF2)‐activating (a master regulator of the cellular antioxidant defense that counteracts lipid peroxidation), or lipid antioxidant strategies to preserve muscle integrity and function with advancing age.

**TABLE 1:** Associations between ferroptosis‐related markers and muscle structure or physical function outcomes in human aging cohorts.

Marker	Ferroptosis axis	Sample/source	Functional outcome(s) reported	Direction vs. poorer function^a^	Evidence strength^b^
Ferritin (Fougère et al. 2024; Ho et al. 2022; Kim et al. 2014; Chen, Chen, et al. 2022)	Iron availability/storage	Plasma/serum (community‐dwelling older adults; clinic cohorts)	Gait speed, chair‐rise, SPPB, handgrip, sarcopenia	U‐shaped: both low and high ferritin linked to worse function. Low ferritin often associates with slower gait/lower SPPB. High ferritin has also been associated with sarcopenia.	Moderate–High (multiple cohort studies)
Transferrin levels and saturation (TSAT) (Fougère et al. 2024; Ho et al. 2022)	Circulating iron availability	Plasma/serum	Gait speed, SPPB, hand grip	Low TSAT (transport‐limited iron) → poorer function.	Moderate
Serum iron (Fougère et al. 2024; Ho et al. 2022)	Circulating iron pool	Plasma/serum	Gait speed, SPPB, hand grip	Lower serum iron associates with sarcopenia and poorer function	Moderate
Labile iron pool (LIP) (Huang et al. 2022; Picca et al. 2020)	Redox‐active intracellular iron	Skeletal muscle biopsy (vastus lateralis)	SPPB components; leg strength/power proxies	Higher muscle LIP associates with poorer muscle quality and performance	Moderate
4‐HNE adducts and MDA (TBARS) (Barrera et al. 2018; Bellanti et al. 2018; Inglés et al. 2014)	Lipid peroxidation	Muscle biopsy; plasma/serum	Gait speed, SPPB, strength (also sarcopenia, frailty – indirect effect on mobility)	Higher levels correlate with poorer function	Low–Moderate
TXNIP (Chaves et al. 2022)	Antioxidant/redox control; inflammasome crosstalk	Muscle biopsy; PBMCs	Mobility metrics; strength	Higher TXNIP associates with poorer function (pro‐oxidant/anti‐thioredoxin)	Low
SLC7A11 (xCT) (Gong et al. 2025; Xu et al. 2025)	Cystine/glutamate antiporter; glutathione supply	Muscle biopsy (RNA/protein); PBMC	Sarcopenia	Lower SLC7A11 (reduced GSH synthesis) associates with poorer function/higher peroxidation	Low
ACSL4 (Stierwalt et al. 2021; Wang et al. 2020)	PUFA‐phospholipid enrichment (sensitizes to ferroptosis)	Muscle biopsy; PBMC	Fat oxidation and fat storage within skeletal muscle	Higher ACSL4 associates with poorer function via increased peroxidizable lipids	Low
Ox stress (Traustadóttir et al. 2012) and antioxidants – GPX4 (Rea et al. 2004)	ROS is a ferroptosis driver, and GPX4 is a Ferroptosis suppressor enzyme	Muscle biopsy	Strength/mobility (inferred)	High‐functioning people had lower ROS. Lower GPX4 associates with poorer function/higher lipid peroxidation	Low

Abbreviations: ACSL4, Acyl‐CoA Synthetase Long Chain Family Member 4; CKD, chronic kidney disease; CRP, C‐reactive protein; GPX4, Glutathione Peroxidase 4; PBMC, peripheral blood mononuclear cells; PUFA, polyunsaturated fatty acids; ROS, Reactive Oxygen Species; SLC7A11 (xCT), Solute Carrier Family 7 Member 11, also known as xCT, which is the cystine/glutamate antiporter subunit system Xc^−^ transporter; SPPB, Short Physical Performance Battery; TBARS, thiobarbituric acid reactive substances; TXNIP, Thioredoxin‐Interacting Protein.

^a^ Direction vs. poorer function reflects the prevailing trend across human studies and should not be interpreted as causal. U‐shaped patterns indicate risk at both low and high extremes.

^b^ Evidence strength graded qualitatively by human sample size, replication across cohorts, direct measurement of performance outcomes, and biomarker specificity (very low → high).

## 6 Ferroptosis in Age‐Related Muscle Disorders

Age‐related iron accumulation occurs in multiple organs, including skeletal muscle, nerves, and the brain, and contributes to oxidative damage that increases the risk of aging‐related conditions such as sarcopenia and neurodegeneration, both of which affect physical function (Zeidan et al. 2021). Here, we will discuss the role of ferroptosis in age‐related disorders that affect physical function.

### 6.1 Sarcopenia

Sarcopenia, a progressive disorder of skeletal muscle characterized by loss of mass and function, markedly increases the risk of losing physical independence (Dos Santos et al. 2017), frailty, disability, and even mortality in older adults (Cruz‐Jentoft et al. 2019). Emerging evidence links ferroptosis mediators in aged skeletal muscle to the onset and progression of sarcopenia. One such mediator, TXNIP (Thioredoxin interacting protein), inhibits the antioxidant thioredoxin system, thereby amplifying oxidative stress and potentially promoting ferroptotic sensitivity (Choi and Park 2023). Notably, TXNIP is overexpressed in multiple tissues, including skeletal muscle, in response to cellular stressors such as iron overload or increased ROS generation, inflammation, and metabolic stress; all hallmarks of aging (Choi and Park 2023; Jiang et al. 2022; Maimaiti et al. 2025). Elevated TXNIP levels observed in aging mice further implicate it in age‐related muscle dysfunction and redox imbalance (Maimaiti et al. 2025). Given its role in oxidative stress, ferroptosis, and cellular senescence, TXNIP upregulation may represent a key molecular link between aging and sarcopenic decline.

In addition, iron accumulation in muscle cells has been shown to induce ferroptosis through p53 upregulation, which in turn downregulates a known ferroptosis gene SLC7A11 (encoding the protein xCT that can support GSH synthesis), leading to the accumulation of lipid peroxides, promoting muscle cell death and accelerating the progression of sarcopenia (Jiang et al. 2022). Beyond ferroptotic signaling, iron overload also impairs mitochondrial biogenesis and promotes loss of both mature myofibers and satellite cells, reducing regenerative potential and increasing vulnerability to atrophy (Yan, Beiling, et al. 2021). Together, these findings position ferroptosis as a plausible mechanism contributing to the pathophysiology of sarcopenia and the subsequent physical functional decline in older adults.

### 6.2 Osteoarthritis‐Related Muscle Atrophy

Osteoarthritis (OA), a leading cause of physical function decline in older adults, is increasingly recognized as a systemic musculoskeletal condition that extends beyond joint degeneration to involve muscle weakness and metabolic dysfunction (Egloff et al. 2014). OA is a degenerative joint disorder characterized by pathological changes, including cartilage lesions, subchondral bone remodeling, and synovial inflammation (Katz et al. 2021).

Studies have investigated the levels of iron in the synovial fluid of individuals with OA and have revealed a strong correlation between iron levels and OA severity (Miao et al. 2022; Ogilvie‐Harris and Fornaiser 1980; Yazar et al. 2005; Zhou et al. 2021). Congruently, multiple studies have reported enhanced levels of lipid peroxidation in synoviocytes, synovial fluid, and cartilage of OA patients (Grigolo et al. 2003; Morquette et al. 2006; Shah et al. 2005), as well as downregulation of antioxidant system activity (Maneesh et al. 2005; Miao et al. 2022; Regan et al. 2008). Further, Miao et al. (2022) demonstrated through transcriptomic, biochemical, and microscopic analyses that ferroptosis contributes to OA progression, with reduced GPX4 expression increasing chondrocyte susceptibility to oxidative stress, promoting extracellular matrix degradation, and exacerbating OA, while ferroptosis inhibitors can offer protective effects, thus supporting the potential association between ferroptosis and OA.

Muscle atrophy around joints is a prevalent physical manifestation of OA and is associated with increased infiltration of macrophages exhibiting a proinflammatory senescence‐like phenotype (Xiang et al. 2025). These macrophages promote ferroptosis in skeletal muscle by releasing iron and inflammatory mediators, thereby exacerbating muscle wasting and joint dysfunction. A recent study by Xiang et al. (2025) demonstrated that macrophages isolated from OA quadriceps induced ferroptosis in human skeletal muscle cells in vitro, and that iron content in OA muscle tissue was significantly elevated compared with healthy controls. Collectively, these findings underscore a mechanistic link between senescence, iron dyshomeostasis, and ferroptosis‐driven muscle atrophy, which may cooperatively accelerate joint degeneration and functional decline in older adults.

### 6.3 Other Conditions

In addition to sarcopenia and OA, ferroptosis is associated with several other conditions that can exacerbate age‐associated muscle and physical function reduction. In chronic kidney disease (CKD), impaired renal clearance and disrupted iron metabolism elevate systemic oxidative stress, promoting ferroptosis in muscle cells (Dounousi et al. 2006). The oxidative burden observed intensifies with disease progression and is strongly associated with skeletal muscle atrophy and reduced physical performance (Alves et al. 2021).

Ferroptosis has also been implicated in several other age‐related and chronic diseases that further contribute to muscle wasting and functional loss. In patients with amyotrophic lateral sclerosis (ALS), depletion of GPX4 has been documented in post‐mortem spinal cords in both sporadic and familial ALS, potentially amplifying denervation‐induced muscle atrophy (Wang, Tomas, et al. 2022). This suggests that ferroptosis may mediate motor neuron degeneration in ALS and represents a potential therapeutic target for disease modification.

Ferroptosis may likewise underlie metabolic dysfunction in type 2 diabetes mellitus (T2DM), contributing to the dysfunction of islet β cells (Sha et al. 2021). Research in mouse models has shown that an increase in lipid peroxides blunts antioxidant defenses and alters mitochondrial morphology and structure, supporting the possibility that ferroptosis participates in β‐cell death (with concomitant impairment in insulin secretion). Additionally, T2DM mice exhibited increased iron concentrations in serum and pancreas, suggesting that iron overload may accelerate the progression of T2DM by promoting muscle atrophy and reducing muscle strength (Li, Jiang, et al. 2020), thereby exacerbating age‐related physical function decline.

Similarly, oxidative stress and ferroptosis in conditions such as facioscapulohumeral muscular dystrophy exacerbate direct skeletal muscle damage, while in neurodegenerative diseases, neuronal ferroptosis indirectly accelerates age‐related muscle decline (Fu et al. 2023; Jakaria et al. 2021; Nakamura et al. 2025).

Ferroptosis has also been increasingly implicated in age‐related cardiopulmonary diseases, particularly pulmonary hypertension, chronic obstructive pulmonary disease (COPD), and heart failure (Bai et al. 2025; Meng et al. 2022; Wang, Chen, et al. 2022), which are strongly associated with systemic and respiratory muscle atrophy and have profound functional decline. In COPD, iron accumulation and heightened lipid peroxidation have been observed in both diaphragm and peripheral skeletal muscle, contributing to respiratory muscle weakness and reduced exercise tolerance (Barnes 2022). Pulmonary hypertension and heart failure similarly exhibit dysregulated iron handling, impaired mitochondrial respiration, and ferroptosis‐related oxidative stress within cardiac and skeletal muscle, processes that collectively diminish oxygen utilization, accelerate muscle wasting, and increase fatigue (Bai et al. 2025; Melenovsky et al. 2017). These conditions therefore illustrate how chronic cardiopulmonary pathology can amplify ferroptotic vulnerability in muscle, further compounding mobility loss and loss of independence in older adults.

In sum, across diverse pathologies, ferroptosis emerges as a shared mechanistic driver of declining muscle mass, strength, and physical function in aging.

## 7 Ferroptosis as a Therapeutic Target

The therapeutic strategies summarized in this section, including iron chelators, lipid peroxidation inhibitors, and lifestyle interventions, collectively target iron dysregulation and redox imbalance that underlie sarcopenia and other age‐related muscle‐wasting conditions and therefore can potentially improve physical function in older adults. By reducing excess iron, limiting ferroptotic stress, and improving mitochondrial and antioxidant function, these interventions highlight the translational potential of correcting iron imbalance to preserve muscle mass and function.

### 7.1 Iron Chelation Therapy

Iron chelators have been shown to reduce iron accumulation and oxidative damage caused by iron overload, mitigating ferroptosis as well as muscle loss in older adults (Qin et al. 2024). By binding excess iron, these agents decrease lipid peroxidation and oxidative damage, protecting against ferroptosis as well as preventing compromised muscle function and recovery (Kardasis et al. 2023). One such iron chelator, deferoxamine, has been shown to be an effective inhibitor of ferroptosis and is approved by the FDA as well as the European Medicines Agency for treatment of patients with iron overload (Hiroko et al. 2019; Zhaoyan et al. 2019). A recent study by Bose et al. (2025) showed that the iron chelator deferiprone reduced serum and muscle iron and protected against loss of mass in gastrocnemius and quadricep muscles in a mouse model of sarcopenia. Another study utilized the iron chelator deferasirox in ovariectomized rats to counteract menopause‐associated iron accumulation and showed reductions in systemic iron levels and oxidative stress markers with enhanced serum antioxidant capacity, all of which could potentially also reduce ferroptosis in skeletal muscle (Honari et al. 2025). Iron chelators thus show promise for treating or preventing age‐related muscle loss (Bose et al. 2025).

The clinical application of iron chelation therapy, however, requires careful monitoring due to potential side effects and the need for individualized dosing. Noteworthy, side effects vary widely depending on the specific iron chelator used, but commonly reported side effects include gastrointestinal pain and discomfort, visual and auditory neurotoxicity, inflammation, and hematological effects such as reduction of erythropoiesis (Entezari et al. 2022). Additionally, a case report from about a decade ago reported proximal muscular atrophy and weakness in twins treated with deferasirox for β‐thalassemia transfusion‐related iron overload (Vill et al. 2016). Notably, this is a scenario distinct from age‐associated iron accumulation, where prolonged chelation may induce systemic iron depletion and impair mitochondrial energy metabolism, underscoring the importance of context‐specific application. Future studies are warranted to optimize the type, dose and duration of iron chelation for maximizing therapeutic efficacy in age‐related muscle function decline with minimal side effects. Also, the feasibility and safety of these interventions in older adults require careful consideration. Age‐associated changes in iron handling, multimorbidity, and polypharmacy may constrain the therapeutic window for iron chelation therapies, increasing the risk of unintended iron depletion, metabolic disruption, or off‐target toxicity. Robust dose‐finding trials and long‐term safety studies will therefore be essential to determine whether ferroptosis‐targeted therapies can be safely deployed in geriatric populations.

### 7.2 Peroxidation‐Targeted Strategies

Targeting lipid peroxidation represents a focused therapeutic strategy to counter ferroptosis‐associated muscle atrophy and functional decline, since lipid peroxidation, rather than generalized ROS generation, is the primary driver of oxidative damage in aging muscle atrophy (Han et al. 2023) and physical function decline. As such, directly targeting lipid peroxidation rather than ROS scavenging represents a specific approach to mitigate ferroptosis‐associated muscle atrophy and improve physical function. Supplementation with radical‐trapping antioxidants (RTAs) has been investigated for its potential to block the formation and propagation of lipid radicals (Ruoxi et al. 2024; Shumin et al. 2023). Various RTAs, such as edaravone or copper(II)‐diacetyl‐bis(*N* ^4^‐methylthiosemicarbazone), have been shown to suppress ferroptosis under pathological conditions (Shumin et al. 2023). Natural RTA compounds, including vitamin E and reduced forms of vitamin K, have also been shown to have anti‐ferroptosis and antioxidant properties, highlighting their potential use as accessible and low‐toxicity interventions (Kajarabille and Latunde‐Dada 2019; Mishima et al. 2022). Results from vitamins E, K, and C (antioxidant capacity) supplementation studies in older adults are inconsistent, where some show modest improvements, while many report no independent benefit (Novelli et al. 1997; Paulsen et al. 2014; Veronese et al. 2019; Wang et al. 2024). This is likely because these vitamins broadly target general oxidative stress rather than the specific lipid peroxidation processes central to ferroptosis, underscoring the need for more targeted redox interventions in muscle aging.

Congruently, reducing lipid peroxidation in animal models has preserved muscle mass and function. Treatment with CMD‐35647, a lipid hydroperoxide–reducing compound, prevented muscle atrophy and maintained contractile function in aged mice (Brown et al. 2025). Furthermore, in a model of denervation‐induced muscle loss, inhibition of the cPLA2 enzyme, which contributes to lipid hydroperoxide production, attenuated oxidative damage and muscle deterioration, while scavenging mitochondrial hydrogen peroxide did not offer any protection against atrophy (Pharaoh et al. 2020; Arc‐Chagnaud et al. 2020; Huang et al. 2022). This suggests a specific role for lipid hydroperoxides in age‐associated muscle atrophy. In humans, a systematic review in older adults found that reducing cellular lipid peroxidation by combining aerobic and resistance exercise with antioxidant supplementation effectively supported muscle function (Wang et al. 2025). Collectively, these findings support the therapeutic potential of lipid peroxidation–targeted strategies to protect skeletal muscle and preserve physical function in aging.

Moreover, targeting specific ferroptosis‐related molecular pathways, particularly those governing lipid peroxidation, central to ferroptotic execution, is a promising therapeutic approach for several pathological conditions. For example, inhibition of ACSL4 by thiazolidinediones (a class of hypoglycemic drugs) can reduce the incorporation of polyunsaturated fatty acids into cell membranes, decreasing susceptibility to lipid peroxidation (Sebastian et al. 2016; Yuan et al. 2016). Also, several proteins have been found to limit the lipid peroxidation process, including GPX4, ferroptosis suppressor protein 1 (FSP1), and guanosine triphosphate cyclohydrolase‐1 (GCH1) (Qian et al. 2024). These proteins are potential targets for gene therapy or pharmacological agents to enhance the detoxification of lipid peroxides and prevent ferroptosis. Further investigation into the precise molecular mechanisms of these inhibitors could pave the way for targeted ferroptosis‐modulating therapies. Notably, the feasibility and safety of peroxidation‐targeted strategies remain to be clarified, particularly regarding long‐term tolerability and potential off‐target effects in older adults.

### 7.3 Non‐Pharmaceutical Lifestyle Interventions

Regular physical activity is a well‐established countermeasure against muscle aging and has been shown to attenuate age‐related decreases in muscle mass, strength, and regenerative capacity (Tang et al. 2025). Exercise has also been shown to regulate iron transport, antioxidant defenses, and lipid metabolism (Tang et al. 2025), which, combined, may play a role in ferroptosis inhibition. One recent study found that Nordic Walking training in older women (67.7 ± 5.3 years of age) significantly reduced blood ferritin concentration, indicating a reduction in body iron stores, which was associated with alleviated risk of various diseases such as cancer and heart disease (Kortas et al. 2015). Additionally, exercise may help counteract age‐associated ferritinophagy impairment and consequent oxidative stress by enhancing antioxidant defenses, promoting iron homeostasis, and maintaining mitochondrial quality control, thereby protecting against the formation of pro‐ferroptotic intracellular environments (Di Lorenzo et al. 2025; Tang et al. 2025; Terink et al. 2018; Plaza‐Florido et al. 2024). Harmoniously, several studies from the University of Florida and others suggest that regular physical activity and sustained muscle use are linked to improved iron homeostasis, reducing oxidative stress and thus potentially protecting against ferroptosis‐related muscle loss during aging (Joseph et al. 2016; Kortas et al. 2015; Picca et al. 2019, 2020; Seo et al. 2008; Ward et al. 2020; Xu et al. 2008; Zelber‐Sagi et al. 2014). Collectively, these findings highlight exercise as a promising strategy to preserve skeletal muscle integrity and support both physical and cognitive function with age (Halon‐Golabek et al. 2019).

Other lifestyle interventions, such as dietary modifications to reduce iron intake, also showed promising results (Vargas‐Vargas et al. 2022). However, age‐related declines in intestinal iron absorption warrant careful consideration when assessing dietary strategies to modulate iron status (Zeidan et al. 2024). In this context, time restricted eating may offer an alternative approach to modulate metabolism and potentially influence systemic and cellular iron homeostasis without directly limiting dietary iron intake (Bensalem et al. 2025; Lin et al. 2024; Wojciak 2013). On the other hand, in vitro studies suggest that reducing n‐6 PUFA ratios in cells could reduce the incidence of ferroptosis, possibly through reducing lipid peroxidation and inflammatory responses (Cho et al. 2025; Liu and Lin 2025), highlighting the need to validate in humans. These dietary interventions may thus potentially complement other therapeutic strategies targeting ferroptosis. Nevertheless, despite encouraging evidence, the specific impact of diet and supplement intake on ferroptosis has not yet been fully elucidated, requiring further research.

## 8 Current Translational Caveats

Despite growing preclinical evidence linking ferroptosis to muscle atrophy and functional decline, several translational barriers remain. First, ferroptosis readouts in human tissues have a low level of specificity. Ferroptosis in rodent models is verified by genetic and ultrastructural indicators (e.g., rescue by ferrostatin‐1, GPX4 depletion, and ACSL4 activation) (Sun et al. 2024), which are challenging to apply in human studies. As such, elevated ferritin, 4‐HNE, or MDA levels, which while informative, can also indicate general oxidative or metabolic stress and are not specific for ferroptotic death. Translation will depend on the development of trustworthy ferroptosis‐specific biomarkers in human muscle, such as acyl‐lipid peroxidation signatures or NCOA4‐mediated ferritinophagy markers. Additionally, direct extrapolation of rodent findings to humans is limited by ultrastructural and pharmacologic criteria that define ferroptosis in rodent models as well as interspecies variations in antioxidant buffering, iron turnover, and muscle composition (type I vs. II fiber dominance). These methodological constraints limit causal inference and underscore the need for human‐validated ferroptosis signatures.

Secondly, most therapeutic studies targeting ferroptosis, including those employing iron chelators or radical‐trapping antioxidants, are based on animal models using high, short‐term doses or administration routes that differ substantially from human pharmacology (Brown et al. 2025; Pharaoh et al. 2020). For instance, the commonly used chelators deferoxamine and deferiprone have limited oral bioavailability and variable skeletal muscle penetration, whereas deferasirox can produce dose‐dependent nephrotoxicity and even paradoxical myopathy at high concentrations (Entezari et al. 2022; Vill et al. 2016). Thus, optimizing dosing regimens and delivery routes in humans remains a key clinical challenge. Moreover, off‐target redox effects complicate interpretation (Entezari et al. 2022). The benefits of chelators and antioxidants in experimental animals may be due to general redox modulation rather than specific ferroptosis blockade, as they may change immune signaling, heme synthesis, or mitochondrial function independently of ferroptosis inhibition. Establishing causality and directing the development of treatments will require longitudinal studies that combine functional phenotyping and direct muscle ferroptosis markers. Refining these therapeutic strategies in human studies will be critical for establishing whether targeted ferroptosis modulation can mitigate muscle atrophy and consequent physical function decline during aging.

Another key translational limitation is the substantial individual heterogeneity inherent to muscle aging, which robustly influences susceptibility to ferroptosis. Interindividual differences in baseline iron status, antioxidant capacity, and systemic inflammation, in addition to lifestyle differences, may impact and shape the threshold at which lipid peroxidation becomes pathogenic (Gutteridge and Halliwell 2000; Ji 2008). At the tissue level, muscles differ markedly in fiber‐type composition, mitochondrial density, and metabolic profile (Hood 2001; Schiaffino and Reggiani 2011), all of which influence iron handling and redox behavior, and may result in muscle‐specific vulnerability to ferroptotic stress. Sex‐dependent differences in iron metabolism, particularly the rise in ferritin and hepcidin after menopause (Kastrati et al. 2024; Milman et al. 1993), further complicate generalization of mechanistic models across populations. Comorbidities common in older adults, including chronic kidney disease, anemia of inflammation, obesity, and diabetes, can themselves alter systemic iron flux, promote oxidative stress, and impair mitochondrial function, thereby amplifying or masking ferroptosis‐related signatures in muscle (Alshwaiyat et al. 2021; Marques et al. 2022; Mehdi and Toto 2009). Together, these sources of biological variability highlight the need for stratified study designs and caution against assuming uniform ferroptotic mechanisms across individuals or muscle groups.

## 9 Conclusion

Ferroptosis is emerging as a possible mechanism driving age‐related skeletal muscle atrophy and physical function decline in older adults. Through iron‐dependent lipid peroxidation, mitochondrial dysfunction, and interactions with chronic inflammation and senescence, ferroptosis may directly compromise skeletal muscle integrity, strength, and regenerative capacity, thus compromising physical function. Evidence from cellular, animal, and human studies increasingly implicates ferroptosis, rather than general oxidative stress, in chronic conditions that jeopardize muscle function in advanced age, such as sarcopenia, osteoarthritis, and other pathologies that impair physical function.

However, critical knowledge gaps remain relating to: (a) the precise triggers of ferroptosis in aging muscle; (b) the interplay between systemic versus local iron dysregulation; and (c) methodological constraints that limit the definitive identification of ferroptotic cell death in human tissues. Primarily, the lack of sensitive, compartmentalized labile cellular iron (key driver of ferroptosis) quantification methods limits the ability to define the exact impact of ferroptosis on aging skeletal muscle cell mass and function. Additionally, the fact that ferroptosis in skeletal muscle is multifactorial, entailing several age‐influenced processes, such as chronic low‐grade inflammation, hormonal changes, and nutritional status, adds an extra layer of complexity. Finally, and critically, the lack of long‐term, longitudinal studies on aging skeletal muscles in humans hinders our ability to understand the effect of ferroptosis‐induced damage on skeletal muscle mass and function. This is because age‐related changes in iron metabolism, antioxidant defenses, and mitochondrial function are dynamic and heterogeneous; thus, single‐time‐point measurements may fail to capture the progression and variability of ferroptosis susceptibility in skeletal muscle. Moreover, inter‐individual biological heterogeneity, including differences in individual iron status, antioxidant capacity, systemic inflammation, muscle fiber composition, sex‐dependent iron regulation, and common age‐related comorbidities, can significantly affect tissue iron levels and antioxidant capacity (Zeidan et al. 2023), thus modulating susceptibility to ferroptotic stress in skeletal muscle, underscoring the need for stratified study designs and caution when generalizing mechanistic models across populations.

Addressing these gaps, while also considering systemic age‐associated physiological changes and individual differences is essential for the development of targeted strategies, pharmacologic or lifestyle‐based, to mitigate ferroptosis, preserve skeletal muscle function, and maintain physical independence in older adults. Bridging this gap requires harmonized human studies that link ferroptosis‐specific molecular signatures with longitudinal changes in strength and mobility. To facilitate this translational progress, three concrete research priorities are proposed in Box 1, outlining immediate steps toward developing standardized biomarkers, mechanistic interventions, and cross‐center methodological harmonization for ferroptosis research in aging muscle.

### 9.1 Synopsis—Knowledge Gap

Importantly, based on currently available evidence, ferroptosis appears to be a plausible mechanism contributing to age‐associated muscle wasting and functional decline. Yet, distinguishing ferroptosis from other forms of oxidative injury in human muscle remains a major barrier, and current biomarkers lack the specificity needed to confirm ferroptotic activity in vivo. Future studies incorporating validated ferroptosis markers will be essential for establishing causality and guiding therapeutic strategies.

BOX 1 ***Future Directions*: Translating Ferroptosis Biology Into Clinical Aging Research.**

Despite strong preclinical evidence linking ferroptosis to muscle atrophy and physical decline, translation to human aging remains limited. Future studies should focus on defining *when*, *where*, and *to what extent* ferroptosis contributes to functional human aging.

1. *Validate a minimal ferroptosis biomarker panel predicting 12‐month functional decline.* Develop and validate a standardized biomarker panel (e.g., ferritin, hepcidin, 4‐HNE adducts, GPX4, ACSL4 expression, and mitochondrial lipid oxidation signatures) capable of forecasting mobility outcomes such as the Short Physical Performance Battery (SPPB) or gait speed. Harmonizing measurement platforms across cohorts will allow multi‐center comparison and enable ferroptosis phenotyping in population studies.
2. *Conduct head‐to‐head interventional pilots targeting ferroptosis mechanisms.* Small mechanistic trials should directly compare a radical‐trapping antioxidant (RTA) versus a clinically approved iron chelator to evaluate effects on lipid peroxidation, mitochondrial function, and muscle quality. Such head‐to‐head studies would clarify mechanistic specificity and inform dose, route of administration, and safety for translation.
3. *Standardize biopsy‐based lipidomics and iron proteostasis assays across centers.* Aging‐related muscle research requires reproducible ferroptosis readouts. Establishing consensus analytical pipelines for tissue lipidomics, ferritinophagy markers (e.g., NCOA4, FTH1), and labile iron quantification will improve reproducibility and bridge the gap between molecular biomarkers and clinical endpoints.

## Author Contributions

R.S.Z. conceived the idea, designed the review framework, and led the planning, writing of the original draft, figure preparation, and manuscript editing. S.R. contributed to writing the original draft and assisted with critical editing and organization. A.P., E.M., C.L., J.F.C., and S.D.A. provided expert review, conceptual input, and manuscript editing. All authors have read and approved the final manuscript.

## Funding

Authors efforts for this work is supported by grant R01AG075136 from the National Institute on Aging for S.D.A. and C.L., a training grant (T32 AG062728) from the National Institute on Aging for R.S.Z., and grants DK134583 and DK137863 from the National Institute of Digestive, Diabetes and Kidney Diseases (supporting J.F.C.). S.D.A. and R.S.Z. were supported by the RC1 core of the Claude D. Pepper Older Americans Independence Center at the University of Florida (P30 AG028740). E.M. recognizes support by intramural research grants from the Università Cattolica del Sacro Cuore (D1.2024 and D1.2025), EU PRIN 2022YNENP3, and NextGenerationEU in the context of the National Recovery and Resilience Plan, Investment PE8‐Project Age‐It: “Ageing Well in an Ageing Society”. This resource was co‐financed by the NextGenerationEU (DM 1557 11.10.2022).

## Conflicts of Interest

The authors declare no conflicts of interest.

## Data Availability

Data sharing not applicable to this article as no datasets were generated or analyzed during the current study.
